# Unraveling Vascular and Parenchymal Microenvironment Changes in Patients with Mixed CAA/AD Pathology: A Spatial Transcriptomic In‐Depth Analysis

**DOI:** 10.1002/alz.087677

**Published:** 2025-01-03

**Authors:** Enrique Chimal Juarez, Nur Jury Garfe, Javier Redding, Juan C Troncoso, Travis S Johnson, Cristian A Lasagna Reeves

**Affiliations:** ^1^ Department of Anatomy, Cell Biology, and Physiology, Indiana University School of Medicine, Indianapolis, IN USA; ^2^ Stark Neurosciences Research Institute, Indiana University School of Medicine, Indianapolis, IN USA; ^3^ Department of Anatomy, Cell Biology and Physiology, Indiana University, School of Medicine, Indianapolis, IN USA; ^4^ Stark Neurosciences Research Institute, Indiana University, School of medicine, Indianapolis, IN USA; ^5^ Johns Hopkins University School of Medicine, Baltimore, MD USA; ^6^ Indiana Biosciences Research Institute, Indianapolis, IN USA; ^7^ Center for Computational Biology and Bioinformatics, Indiana University School of Medicine, Indianapolis, IN USA

## Abstract

**Background:**

Cerebral amyloid angiopathy (CAA), defined as the accumulation of amyloid in cerebral blood vessels causing alterations in the blood brain barrier (BBB) and the gliovascular unit, occurs in over 85% of Alzheimer’s disease (AD) cases, positioning CAA as one of the strongest vascular contributors to age‐related cognitive decline. However, the specific mechanisms in the microvasculature that become altered due to amyloid deposition and its downstream effects on the brain are complex and incompletely understood. A spatial transcriptomic analysis comparing pathways affected in the gliovascular niche differently in the presence of vascular amyloid could provide critical insight into the mechanisms underlying cerebrovascular changes involved in the deposition of Amyloid in the cerebrovasculature.

**Method:**

Using NanoString’s GeoMx Human Whole Transcriptome Atlas, which measures over 18,000 protein‐coding genes at each region of interest (ROI) in tissue sections, we evaluated mixed CAA/AD pathology patients. We evaluated and performed selected pair wise comparisons between 4 types of ROI: **1)** *Astrocytes surrounding vascular amyloid*, **2)** *astrocytes surrounding amyloid‐free vasculature*, **3)** *astrocytes surrounding parenchymal amyloid, &* **4)** *astrocytes in an amyloid‐free parenchymal zone*.

**Result:**

Conducting pairwise comparisons among the four types of Regions of Interest (ROIs) unveiled distinctive transcriptomic signatures across ROI categories. Notably, gene expression profiles in regions of vasculature positive for Aβ‐amyloid differed significantly from those in amyloid‐free vasculature, showcasing pronounced gene expression changes. While the signatures corresponding to both Parenchymal amyloid and vascular amyloid have a similar transcriptional signature, they differ in certain pathways. Through meticulous data mining, we identified a co‐expression cluster of genes intricately linked to vascular amyloid deposition. Further analysis involved determining Differentially Expressed Genes (DEGs) based on ROI types, yielding a comprehensive list of potential targets indicative of the perturbations induced by vascular amyloid deposition versus parenchymal amyloid deposition.

**Conclusion:**

In summary, the identified differential (parenchymal vs vascular) genes underscore a clear association with alterations in the neurovascular microenvironment, indicating a discernible shift in vasculature dynamics attributed to amyloid presence. This observation emphasizes the significance of comprehending the changes within the vascular unit to address Cerebral Amyloid Angiopathy (CAA) thoroughly to develop comprehensive strategies to tackle CAA‐related challenges.

